# Overexpression of *GbRLK*, a putative receptor-like kinase gene, improved cotton tolerance to *Verticillium* wilt

**DOI:** 10.1038/srep15048

**Published:** 2015-10-08

**Authors:** Zhao Jun, Zhiyuan Zhang, Yulong Gao, Lei Zhou, Lei Fang, Xiangdong Chen, Zhiyuan Ning, Tianzi Chen, Wangzhen Guo, Tianzhen Zhang

**Affiliations:** 1 National Key Laboratory of Crop Genetics & Germplasm Enhancement, MOE Hybrid Cotton R&D Engineering Research Center, Nanjing Agricultural University, Nanjing 210095, Jiangsu province, China

## Abstract

*Verticillium dahliae* is a causative fungal pathogen and only a few genes have been identified that exhibit critical roles in disease resistance and few has shown positive effects on the resistance to Verticillium wilt in transgenic cotton. We cloned a receptor-like kinase gene (*GbRLK*) induced by *Verticillium dahliae* (VD) in the disease-resistant cotton *Gossypium barbadense* cv. Hai7124. Northern blotting revealed that the *GbRLK* was induced by VD at 96 h after inoculation. The functional *GbRLK* is from D subgenome since a single base deletion results in a frameshift or dysfunctional homologue in the A subgenome in tetraploid cotton. To verify the function of *GbRLK*, we developed the overexpression transgenic *GbRLK* cotton and *Arabidopsis* lines, and found that they all showed the higher resistance to Verticillium in the greenhouse and field trial. The results of the expression profile using transgenic and non-transgenic *Arabidopsis thaliana* revealed that the *GbRLK* regulated expressions of a series genes associated with biotic and abiotic stresses. Therefore, we propose that the increased resistance to *Verticillium dahliae* infection in transgnic plants could result from reduction in the damage of water loss and regulation of defense gene expression.

Receptor-like protein kinase (*RLK*s) form a large multi-gene family in plants, at least 610 members in *Arabidopsis* and about 1131 members in rice^1^. For many of these *RLKs*, only fewer than 2% of the totals RLKs have been identified, and one or a small number of proteins have been analyzed in detail^2^. *RLKs* participate in a diverse range of processes and can be divided into two broad categories. The first category involves in the control of plant growth and development under normal growth conditions, like brassinosteroid insensitive 1 (*BRI1*)^3^,^4^, *CLAVATA1*^5^, and *HAESA*^6^, and the second category in disease resistance and stress responses^7^. *RLK* family members have been implicated in controlling defense or in plant-microbe interactions including tomato *Pto*^8^, rice *Xa21*^9^ *Arabidopsis FLS2*^10^ and Wheat *LRK10*^11^. In *Arabidopsis*, two *RLK*s, AtRLP52 and AtRLP30, play important roles in resistance to the powdery mildew fungus *Erysiphe cichoracearum* and the bacterium *Pseudomonas syringae* pv phaseolicola, respectively^12^,^13^. *AtRLP51* was found to regulate defense against the downy mildew pathogen *Hyaloperonospora arabidopsidis* and *P*. *syringae* pv tomato^14^. Beside the resistance to biotic, several other *RLK* genes are implicated to take part in the signal transduction in abiotic stresses responses. A receptor-like cytosolic kinase gene, *ARCK1*, whose expression is induced by abiotic stress, negatively control ABA and osmotic stress signal transduction^15^. In *Arabidopsis thaliana*, a receptor-like kinase, *GHR1*, mediates ABA- and hydrogen peroxide-regulated stomatal movement^16^. Another example, overproduction of the membrane-bound receptor-like protein kinase 1, *RPK1*, enhance abiotic stress tolerance^17^,^18^.

Cotton (*Gossypium* spp.) is widely cultivated for the important economic value of its fiber. Cotton *Verticillium* wilt caused by *Verticillium dahliae* which is a soil-borne vascular disease^19^. Verticillium wilt, one of the most devastating plant diseases worldwide, is a major concern for cotton producers and triggers severe yield loss each year. This pathogen is particularly difficult to control in cotton. So far, the genetic and molecular mechanisms underlying cotton resistance to *Verticillium* infection are poorly understood and the conventional breeding for improvement of cotton resistance to Verticillium wilt has not been successful^20^. Recently, some efforts have been made to understand the molecular mechanism of the Verticillium wilt disease caused by *V. dahliae*. The *Verticillium* resistance genes, *Ve1*, were cloned by map-based cloning strategy in tomato^21^,^22^,^23^. Zhang *et al.*^24^ cloned and characterized a *Verticillium* wilt resistance gene (*GbVe*) from cotton and analyzed its function in *Arabidopsis thaliana*. Veronese *et al.*^25^ identified a dominant locus in *Arabidopsis* ecotype C-24, *Verticillium dahliae*-tolerance 1 (*VET1*), conferring increased tolerance to *V. dahliae* infection. Vallad and Subbarao^26^ studied infection and colonization process of *Verticillium dahliae* in lettuce cultivars using a green fluorescent protein. Some results show that the phenylpropanoid pathway plays a critical role during the plant defense response to *V. dahliae*^27^,^28^. Several studies have characterized transcriptomic changes in the cotton defence response by deep-sequence approaches^29^,^30^. Xu *et al.*^29^ reported that lignin metabolism has a central role in the resistance of cotton to *Verticillium dahliae* as revealed by RNA-Seq-dependent transcriptional analysis and histochemistry. Using a VIGS approach, several genes were shown to be required for resistance to *V. dahliae* in tomato and cotton by gene silencing pathway^22^,^31^.

Some protein kinases have been isolated in cotton, but they have been shown to function mainly in regulating cotton fiber development. To our knowledge, protein kinase genes associated with cotton *Verticillum* wilt have not been documented yet. The RLK gene (*GbRLK*) in this study is previously cloned among a group of expressed resistance gene analogs that are induced by *Verticillium dahliae* (VD) in the disease-resistant cotton *Gossypium barbadense* cv. Hai7124^32^. However, its expression is also induced by exogenously supplied abscisic acid (ABA), salicylic acid, methyl jasmonate, mock drought conditions and high salinity. Transgenic *Arabidopsis* with constitutive overexpression of *GbRLK* exhibited a reduced rate of water loss in leaves *in vitro*, along with improved salinity and drought tolerance and increased sensitivity to ABA compared with non-transgenic Col-0 *Arabidopsis*^33^. In this study, we analyzed the potential function of *GbRLK* in resistance to *V*. *dahliae*. The isolation of *GbRLK* gene will facilitate the investigation of the molecular basis of *GbRLKs* in resistance, and could open new avenues for breeding cotton varieties highly resistant to the fungal pathogen *V*. *dahliae*.

## Results

### Temporal and spatial expression and genomic evolution analysis of *GbRLK*

In our previous report, *GbRLK* was isolated among 21 RGA clones from *G. barbadense* cv. Hai7124 using degenerate primers designed from the sequences of the conserved domains of receptor-like protein kinases. The cDNA was 1536-bp in length and the predicted ORF starts at nucleotide 330 and ends at nucleotide 1409, encoding a predicted protein of 359 amino acids^32^,^33^. The expression of *GbRLK* was up-regulated by infection of *V. dahliae* strain VD8 at 96 hours, but not in the untreated control or the other time points assayed (Figure S1a), suggesting its potential role in resistance of Hai 7124 to VD.

**Figure 1:**
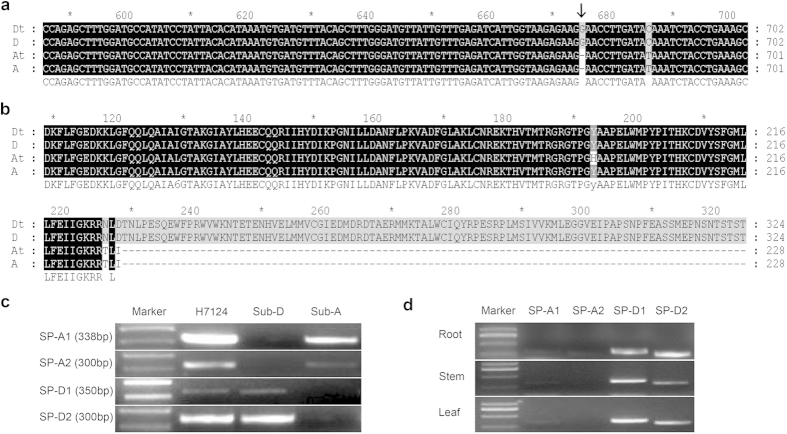
Gene expression and genomic evolution analysis. **(a**,**b**) Comparison of nucleotide (**b**) and predicted GbRLK amino acid (**b**) sequences. A: *G. herbaceum*; D: *G. raimondii*; At and Dt: the At and Dt sub-genomes of Hai7124, respectively. The arrow shows the position where a base is deleted. (**c**) The amplification results using primers specific for the At and Dt sub-genomes. A: *G. herbaceum*; D: *G. raimondii*; H7124: Hai7124. (**d**) The expression of *GbRLK* in root, stem, and leaf 96 h after inoculation with VD8.

Southern blotting showed that the genome of allotetraploid cotton contained two to three copies of the *GbRLK* gene (Figure S1b). We isolated the full-length of *GbRLKs* from two diploid species, A-genome *G. herbaceum* and D-genome *G. raimondii*, and in the A and the D subgenomes in allotetraploid cotton, Hai7124. Sequence analysis indicated that the orthologous gene from the A-genome *G. herbaceum* and the A subgenome in tetraploid cotton both have a single base pair (bp) deletion that is predicted to cause premature termination (Fig. 1a,b), suggesting its defunctionalization before the formation of tetraploid cotton. We search the predicted amino acid sequence of GbRLK in NCBI, and found GbRLK protein from the D subgenome of Hai7124 shares a highly conserved complete serine/threonine kinase and tyrosine kinase domain (26 ~ 310), but GbRLK protein from the A subgenome just shares a partial serine/threonine kinase domain (Figure S2). Meanwhile, we predicted amino acid sequence of GbRLK with SMART and found GbRLK protein from the D subgenome possess a low complexity domain (317–341) compared to one from the A subgenome. Using the subgenome-specific PCR primers (SP-A and SP-D) (Table S1), we confirmed that the expression of *GbRLK* was from the D subgenome (Fig. 1c,d). So the functional *GbRLK* may originate from the D subgenome in tetraploid cotton.

**Figure 2:**
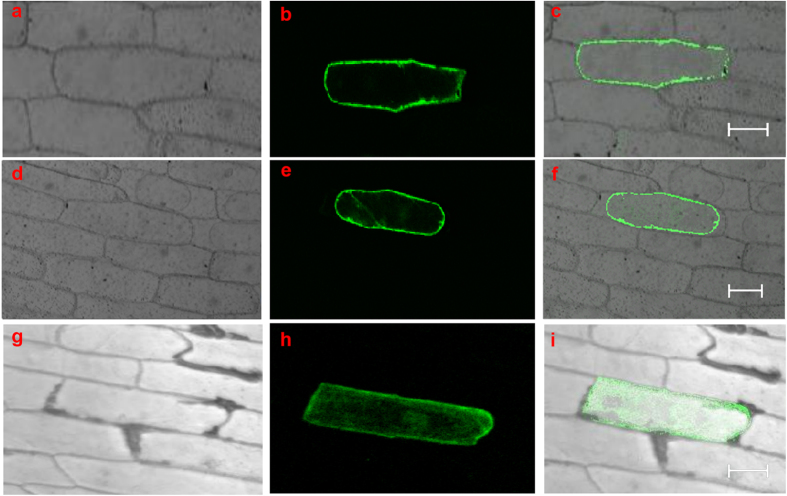
Subcellular localization of the GbRLK proteins in onion epidermal cells. The fusion construct of the GbRLK-GFP and the GFP control plasmid were transformed into onion (*Allium cepa*) epidermis cells by biolistic bombardment. (**a**–**c**) Localization of 35S-*GbRLK*-*GFP*; (**d**–**f**) The cell wall and plasma membrane were separated by treatment with 20% sucrose for 30 min; (**g**–**i**) Localization of *35S-GFP* (positive control); (**a**,**d**,**g**) Onion cell under bright field; (**b**,**e**,**h**) Onion cell under 488 nm excitation light; (**c**,**f**,**i**) GFP in the onion cell of overlayed images. Bar = 100 um.

To examine its subcellular localization, we fused the *GbRLK* coding region in frame with the N-terminus of the green fluorescent protein (GFP) coding region under control of the CaMV 35S promoter. Onion epidermal cells were separately transformed with either the *35S::GbRLK-GFP* fusion or the *35S::GFP* plasmid control by biolistic particle delivery (Fig. 2). GFP-specific fluorescence was evident in the membrane in cells transformed with the fusion vector (Fig. 2a–c). If the cell wall and cell membrane were separated by treatment with 20% sucrose for 30 min, GFP fluorescence was also observed in the membrane (Fig. 2d–f), whereas in control cells transformed with the 35S::GFP plasmid, GFP fluorescence was detected throughout cells (Fig. 2g–i), indicating that the *GbRLK* was localized mainly in the plasma membrane.

### *GbRLK* overexpression improved tolerance to *Verticillium dahliae* in transgenic *Arabidopsis*

**Figure 3:**
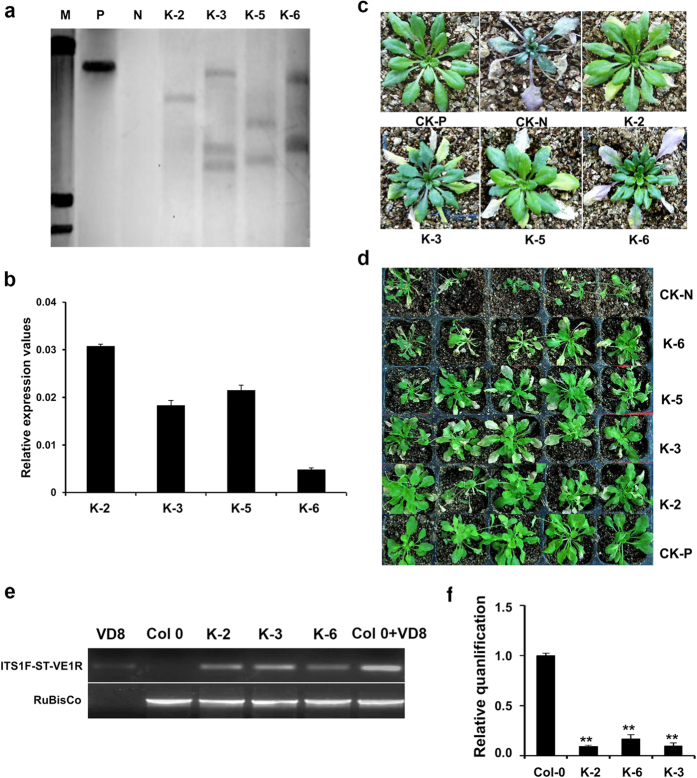
The 35S::*GbRLK* transgenic lines improved the resistance to VD isolate VD8 and BP2. (**a**) Southern blot analysis of four independent T5 generation homozygous transgenic lines. P: positive control pBI121, N: non-transformed plant; transgenic lines K-2, K-3, K-5, and K-6. (**b**) C: qRT–PCR analysis of *GbRLK* expression in homozygous transgenic *Arabidopsis* lines. (**c**,**d**) Phenotype of *Arabidopsis* at 4 weeks after inoculation. CK-P, mock-inoculated wild-type Col-0. CK-N, wild-type Col-0 inoculated with VD. (**e**) Detection the biomass of the *Verticillium dahlia* in the transgenic plant with RT-PCR by comparing V. *dahliae* internal transcribed spacer (ITS) transcript levels (as a measure for fungal biomass) relative to *Arabidopsis* Rubisco transcript levels (for equilibration) at 14 d post-inoculation. (**f**) Detection the biomass of the *Verticillium dahliae* in the transgenic plant with Quantitative real-time PCR. The relative average fungal biomass is shown with standard errors. Asterisks indicate significant differences when compared with colonization of the wild type Col-0. The average fungal biomass was determined using at least six *Verticillium*-inoculated plants for each genotype. *V. dahliae* biomass quantificated in plants as described by Ursula Ellendorff^46^.

The 35S::GbRLK construct was introduced into *Arabidopsis* ecotype Col-0 by the floral dip method^34^. Subsequently, four positive transgenic homozygous lines were confirmed by PCR of *NPT*II and 35S-GbRLK and designated as K-2, 3, 5 and 6 (Figure S3a). Southern blotting demonstrated that these lines were independent transformants, and each carried between one and three copies of the *GbRLK* genes (Fig. 3a). The qRT-PCR revealed that the *GbRLK* gene was expressed in all four transgenic lines, and that expression was highest in the K-2 (Fig. 3b). There was no consistent difference between the transgenic *Arabidopsis* and the control as measured in fresh weight (Figure S3b).

Two types of VD isolates, the highly virulent defoliating isolate VD8 and the medium virulent non-defoliating isolate BP2, were used to inoculate the transgenic *Arabidopsis* to assay their resistance to *Verticillium* wilt. Leaf tissues of the transgenic and wild-type (WT) *Arabidopsis* plants began to show the same symptoms after inoculation with the VD8 after two weeks. Although the inoculated plants showed typical disease symptoms with wilt and chlorosis, a distinct difference was observed between the WT and transgenic plants after four weeks (Fig. 3c). In the transgenic plants, infection resulted in gradual wilting, senescence, eventual defoliation, but not death; however, in the WT, stunting, and in some cases a sudden general wilt and death occurred. Additionally, there were also significant differences between the transgenic and the WT plants in fresh weight (FW), anthocyanin accumulation, chlorophyll content and level of leaf chlorosis (Table 1). Less than 40% chlorosis could be observed in the transgenic lines as compared to more than 60% in the WT. Among these transgenic lines, K-2 showed the highest resistance, consistent with the expression level of the *GbRLK*. The same relative differences in resistance between the transgenic and WT plants inoculated with the BP2 were observed (Fig. 3d). It is concluded that the transgenic *Arabidopsis* lines are more tolerant to VD infection compared to the WT.

To quantify *V. dahliae* colonization in the transgenic lines and wild-type, the *V. dahliae* strain VD8 biomass was measured with real-time RT-PCR. Determination of the average fungal biomass revealed significantly inhibited fungal colonization in *V. dahliae*-inoculated the transgenic lines K-2, K-3 and K-6 when compared with the inoculated Col-0 plants, since at least five times the amount of fungal biomass was detected in Col-0 plants at 2 weeks post-inoculation. The result revealed that overexpression of *GbRLK* inhibited the fungal spores spread in plants (Fig. 3e,f). These results showed that overexpression of *GbRLK* conferred tolerance to VD, and that *GbRLK* may function as a positive regulator in plant responses to VD infection.

### *GbRLK* overexpression lines increased cotton resistance to *Verticillium* wilt in greenhouse and field

**Figure 4:**
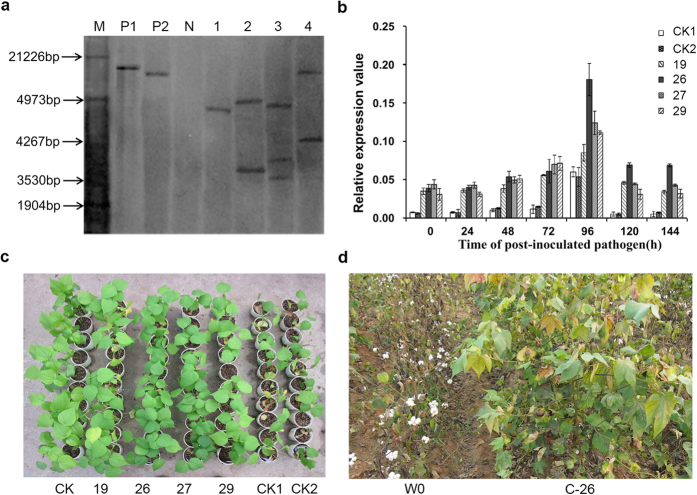
Resistance phenotypes of independent T4 generation homozygous transgenic cotton lines. (**a**) Southern bolt analysis of four independent T4 generation homozygous transgenic lines. Genomic DNA was digested with *EcoR* I and hybridized with a 750 bp fragment of the NPTII gene. M: molecular weight marker; P: positive control pBI121; N: non-transformed plant W0; Transgenic cotton lines C-19, C-26, C-27, and C-29. (**b**) Relative expression levels of *GbRLK* at 0, 24, 48, 72, 96, 120, and 144 h post-inoculation in transgenic lines (C-19, C-26, C-27, C-29), wild type (W0) and W0 transformed with the empty vector pBI121 without *GbRLK* gene. Error bars represent SE, n = 3. (**c**) CK: the non-transgenic plant W0 inoculated with water. CK1: the control transformed empty vector pBI121without *GbRLK* gene inoculated with VD; CK2: the non-transgenic plant W0 inoculated with VD. Similar results were obtained from other three independent experiments. (**d**) Resistance phenotype of independent transgenic cotton line C-26 in Xinjiang province, China in 2014.

The 35S::GbRLK construct was further introduced into cotton (W0 line) by *Agrobacterium*-mediated transformation as previously described^35^. The positive transgenic plants were confirmed by PCR of *NPT*II and 35S-GbRLK. and four transgenic homozygous lines designated C-19, C-26, C-27 and C-29 were developed as the same procedure as in *Arabidopsis* (Fig. 4a,b and Figure S4). There were no significant differences between height of plants, number of fruit branch and boll number of per plant and single boll weight and yield for lines C-19, C-26, C-27 and C-29, and W0 in no *V. dahliae*-inoculated field (Table S2).

VD isolate V991, a highly virulent defoliating isolate from the Yangtze River cotton growing region in China, was chosen to evaluate their resistance to VD in greenhouse for two years (Fig. 4c). The homozygous transgenic lines showed cotyledon yellowing and defoliation with no apparent resistance to VD compared with the control at early period after inoculation. However, as the plants grew and the fungus continued to multiply, the transgenic cotton had better performance and increased resistance to the pathogen as compared to the control. At this time, the non-transgenic controls began to show defoliation of the true leaves, stem browning, and some plants eventually died. The disease index was as high as 56%. Leaves of the transgenic cotton plants showed yellowing, and some abscised and fell off, but there was almost no plant death as compared to W0. The experiment conducted in 2012 was similar to that in 2011 except for a higher disease incidence ratio than in 2011 (Table 2).

To assess the resistance of transgenic lines against *Verticillium* wilt in field, we planted seeds of four transgenic lines, C-19, C-26, C-27 and C-29, and non-transgenic W0, and the resistant cultivar *G. barbadense* cv. Hai7124 were in a natural pathology nursery in Xinjiang and Henan, China in 2014 cotton-growing seasons. For the resistance to *Verticillium*, the transgenic lines C-19, C-26, C-27 and C-29 showed lower disease indexes and dead rate compared to non-transgenic W0 (Table 3, Fig. 4d). This study confirmed our transgenic Arabidopsis conclusion and further demonstrated that the overexpression of *GbRLK* in transgenic cotton plants can provide substantial resistance to *Verticillium* wilt resistance.

Four transgenic lines, C-19, C-26, C-27 and C-29 plants produced more seed cotton yield per 667 m_2_ than W0 in the *Verticillium* field trials. A significant difference was observed between the transgenic lines C-19, C-26, C-27 and C-29 and its receptor W0 in the height of plants, number of fruit branch and boll number of per plant, lint percent and single boll weight except lint percent.

### *GbRLK* overexpression changed the transcriptomic profile in transgenic *Arabidopsis*

**Figure 5:**
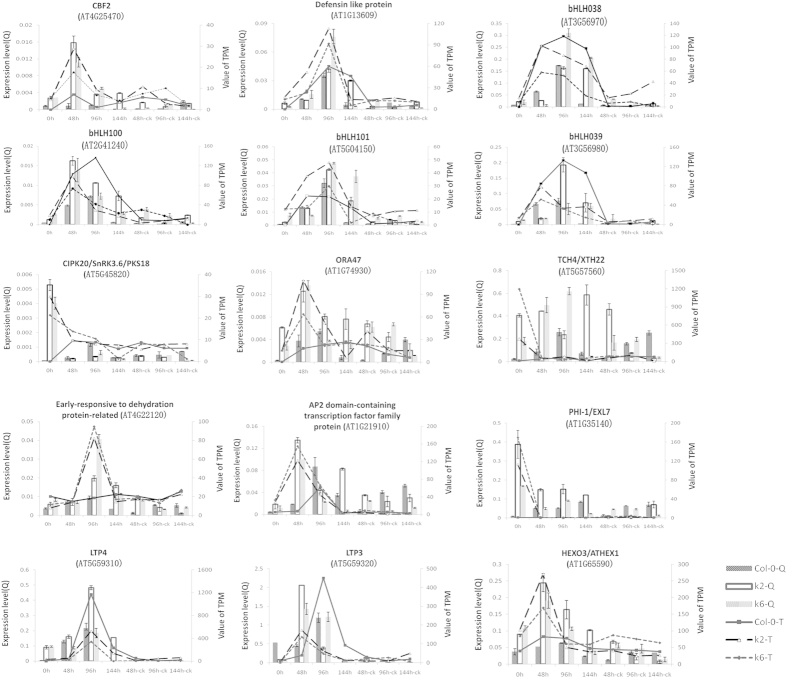
qRT–PCR validation the results of expression profile. Column represents the result of qRT–PCR, Q means qRT–PCR; Line represents the result of sequencing, T means the value of TPM (transcripts per million mapped read).

In order to reveal how the expression of *GbRLK* improved the tolerance to stresses in transgenic *Arabidopsis thaliana*, their gene expression profiles were studied. We used the two homozygous transgenic lines K-6 and K-2 different in their resistance to VD, and Col-0 as WT control. Expression of differentially expressed genes behaved similarly between qRT–PCR and deep-sequencing methods (Fig. 5 and Table S3).

Differentially expressed genes between the transgenic and its WT plants gave some clues to the molecular events related to the *GbRLK* gene resistance mechanism. There were 18 genes including six transcription factors (Table S4) which were detected only in the two uninoculated transgenic lines K-6 and K-2. We also found 35 up-regulated transcription factors, of them; five were related to water stress, six to pathogenic bacteria infection, four to water and pathogen stress (Table S5).

After VD inoculation, there were 227 genes that were specifically up-regulated in the two transgenic lines but not in the WT; of these, there were 198, 26 and 3 specifically up-regulated at 48, 96 and 144 h post-inoculation, respectively. The 227 specifically up-regulated genes could be classified into 96 functional categories related to hormone signal pathways, salt stress etc by gene ontology (GO) enrichment analysis (Table S6). There were 17 genes related to abscisic acid and oxidation reduction (Table S7). This was not surprising given the well established role of ABA in plant defense responses to *Verticillium* wilt. GO enrichment analyses 144 h post-inoculation, showed only one gene, peroxidase 71 (AT5G64120), which participated in five biological processes containing defense response to fungus infection and response to oxidative stress etc (Table S6).

These results revealed that the transcriptomic profiles were altered in transgenic lines compared to the WT. When expressed in transgenic *Arabidopsis*, *GbRLK* could regulate the expression of many genes related to stresses, which may in turn result in improvement of the tolerance to *Verticillium* in transgenic *Arabidopsis*.

## Discussion

Several kinase family members have been implicated in controlling disease resistance or in interacting with proteins of microbial origins. *GbRLK* was isolated from Hai7124, a widely used *Verticillium* wilt*-*resistant cultivar and expressed at 96 h post-inoculation with VD8 isolate, suggesting that *GbRLK* may play a role in defense against VD infection in cotton. In this study, we have not analyzed whether the expression of *GbRLK* leads to activation of general stress or results in a specific pathogen resistance yet, but we found many up-regulated genes related to abiotic stresses in comparing the transcriptional profiles between the transgenic and non-transgenic *Arabidopsis*, and our results showed that *GbRLK* was related to abiotic stresses^32^. We also found the expression of *GbRLK* was detected just 96 h after inoculation but not at any other time point. It is supposed that *GbRLK* may be a member of signal pathways, its transcriptional level will decline rapidly when it recognizes the signals and activates downstream pathways since it needs a certain time for *Verticillium* invading into cotton plant.

Phylogenetic analysis give us some message about the classification and function of *GbRLK*^32^. AtPR5K contain thaumatin-like domains and the extracellular domain of PR5K (PR5-like receptor kinase) is most highly related to acidic pathogenesis-related protein 5 2. *TaLRK10* was isolated from hexaploid wheat by using a combination of subgenome map-based cloning. It encodes a CC-NBS-LRR type of protein and overexpressed wheat plants enhanced resistance to leaf rust^11^. Based on the extracellular domains of RLKs, we know that *AtPR5K* and *TaLRK10* belong to different subclass RLK proteins, so phylogenetic analysis cannot give a clear clue that *GbRLK* was which subclass of RLK proteins, but it indicated the GbRLK protein may share the resemble function with *AtPR5K* and *TaLRK10*. Meanwhile, the results of phylogenetic analysis using cotton kinase proteins suggested *GbRLK* may be a new functional receptor-like kinase protein in cotton genome.

Salicylic acid (SA), ethylene (ET) and jasmonic acid (JA) are synthesized and activate distinct defense pathways involved in complex defense signaling networks^36^. In our previous study, *GbRLK* transcription was induced by exogenously supplied salicylic acid and methyl jasmonate^32^. In this study, the differentially-expressed genes contained many ones that are related to JA and SA. Our results showed that *GbRLK* also has profound roles in modulating diverse plant-pathogen interactions mediated at least in part by cross talk with the JA and SA biotic stress signal pathways. Meanwhile, the results of the transcriptomic profiling revealed that most of the up-regulated genes related to disease resistance were responsive to chitin. This suggested that the transgenic *Arabidopsis* plants showed improved resistance to VD by regulating the chitin response signaling pathway.

Abiotic stress has a strong effect on ABA accumulation and is known to influence plant resistance to pathogens. The role of ABA in pathogen defense is, so far, poorly understood and even controversial, however. Several reports have shown that ABA plays an important role in pathogen defense^37^,^38^,^39^. In our previous report, we report that *GbRLK* taken part in abiotic stress signaling pathways and induced by exogenously supplied abscisic acid (ABA), mock drought conditions and high salinity. Transgenic Arabidopsis with constitutive overexpression of *GbRLK* showed high tolerance to abiotic stresses^32^. Lignification of the cell wall, formation of lignitubers, and the restriction of vascular spread by occlusion are all common physiological responses in plants against VD infection^40^; these responses not only restrict the pathogen from spreading further, but also restrict water transport within the vascular system. *Verticillium* infects plants *via* the roots and enters the xylem, where it releases conidia that spread upwards through the vessels with the transpiration stream^19^. Pathogen attack changes the infected plant’s water balances or fluxes and causes severe dehydration stress. In our study, we found no direct evidence of a relationship between ABA and *Verticillium* infection. But we found that proliferation of VD was inhibited in transgenic *Arabidopsis thaliana* expressing *GbRLK*, and the expression profile revealed that overexpression *GbRLK* regulated the expression of many genes related to stress, especially these genes related to abscisic acid (ABA) stimulus. For example RD26, a dehydration-induced NAC protein, RD26, is involved in a novel ABA-dependent stress-signaling pathway. It is possible that RD26 acts upstream of ABA signaling pathway and is positively regulated by *GbRLK* under VD stress in plants. Thus, we can speculate that one reason for the improvement of resistance to VD may be that the *GbRLK* gene activated the ABA signaling pathway under VD stress, decreasing water loss, and subsequently inhibited VD spread within the host.

*GbRLK* may participate in a plant resistance pathway. Sequence analysis revealed that the promoter region contains many cis-acting stress-responsive elements such as W-Box, MYB-core, W-Box core, TCA-element and others. We constructed a vector containing a 1,890-bp sequence in the 5′ region upstream and transformed it into Arabidopsis thaliana. GUS histochemical staining analysis showed that *GbRLK* was induced by *Verticillium dahlia*^32^. The transcriptomic profiling analysis showed that the approximately two-third up-regulated transcription factors were associated with abiotic and pathogen stresses. The results of search up-regulated gene biological processes in NCBI database revealed that the number of genes related to oxidation reduction was the largest. The generation of reactive oxygen species (ROS) is a common component of disease resistance responses^41^. In our results, 17 genes related to oxidation reduction included 6 cytochrome P-450 family proteins, a NAD(P)-linked oxidoreductase-like protein. These results suggested that overexpression *GbRLK* activate the ROS signal in transgenic. Although the mechanism of *GbRLK* ptomote ROS production is not clear, VD infection may elicit a biphasic ROS accumulation in transgenic plants. Besides, in all ten transcription factors related to disease resistance, seven were response to chitin, indicated that *GbRLK* may participate in a plant resistance pathway involved in the chitin. It is interesting to note that four bHLH genes were specific genes in transgenic plants and were accompanied by a variation with time of VD infection and these 4 bHLH genes in this study involved in Fe iron deficiency stress^42^, this results indicated that *GbRLK* may be involved in the signal transduction processes during ion stress or involved in the *Verticillium dahlia* invasion process.

In conclusion, our genetic and transgenic analyses identified a receptor-like kinase, *GbRLK*. The transcriptomic profiling analysis showed the *GbRLK* gene may participate in various transmission response signals. Overexpression of *GbRLK* improved the resistance against VD through at least two independent mechanisms: 1) regulation of defense gene expression; and 2) activation of the signaling pathway about ABA, resulting in decreasing transpiration and subsequently inhibiting the spread of VD. Based on our results, we thought it will be wise to select right cultivar as receptor in transgenic breeding to improve the resistance to *Verticillium dahliae*, for example, we can transform the resistance disease gene to cultivar with high resistance to drought, or transform right gene which may be a junction of biotic and abiotic stresses signal pathways. Cloning of the *GbRLK* gene will not only facilitate investigation of the molecular basis underlying *Verticillum* wilt but also provide a resistance gene for the future genetic engineering of novel cultivars resistant to the deadly fungal pathogen *V. dahliae*.

## Materials and Methods

### Plant materials, growth conditions and inoculation treatments

Seeds of *G. barbadense* cv. Hai7124 were delinted with sulfuric acid. To ensure that the seeds were free of pathogens, they were subjected to 37% formaldehyde fumigation for 24 h. The treated seeds were sown and grown in pots filled with sterile soil at 22–27 °C. VD was cultured in 100 ml Czapek’s liquid medium in 250 ml flasks for 8–10 d at 25 °C with shaking. The VD culture was then filtered through four layers of cheese cloth and the number of spores counted under a microscope and adjusted the final spore concentration was adjusted to 1 × 10^8^/ml before inoculation.

Seedlings with two and one heart-shaped leaves of Hai7124 were inoculated with VD spore suspension. The detailed procedures of surface-sterilization, growth conditions of *Arabidopsis* seeds, inoculation, phenotypic evaluation and VD culture were performed as described by Veronese *et al.*^25^

### DNA sequence, Southern blotting and expression analysis

*GbRLK* gene homologs were amplified from *G. herbaceum*, *G. raimondii*, and *G. barbadense* Hai7124 genomic DNA with specific primers (GbRLK1-F/R)(Table S1), using high-fidelity ExTaq DNA polymerase (TakaRa Biotech (Dalian) Co, Ltd, China). The PCR products were subcloned into the pMD18-T vector (TaKaRa, Japan) and sequenced. In order to obtain the sequence from the At- and Dt-subgenomes, at least 10 clones from the tetraploid species, Hai7124, and three clones from the diploid progenitors were sequenced.

Southern blotting was conducted in transgenic *Arabidopsis* and the cotton cultivar Hai7124 using the 3′ 407-bp sequence of the full-length cDNA amplified using *GbRLK*3-F/R primers (Table S1) as a probe. A 750 bp fragment of the *NPT*II coding region which was amplified (NPTII-F/R) (Table S1) was used as a probe in cotton. Hybridization was conducted according to the instructions of the DIG High Prime DNA labeling and Detection Kit (Roche, Switzerland).

Specific primers (GbRLK2-F/R) (Table S1) were designed to evaluate the expression level of *GbRLK* by qRT-PCR as described previously^32^ using the ABI 7500 Real Time System (PE Applied Biosystems, USA). *EF1a* (At5g60390) (EF-F/R) (Table S1) from cotton and the gene (AtRuBisCo-F3/R3) for the RuBisCo large subunit (Table S1) which generate a 120 bp amplicon in *Arabidopsis* were used as internal gene standards. Northern blotting was performed as described by Yamaguchi-Shinozaki and Shinozaki^43^.

### Subcellular localization of GbRLK

The full length *GbRLK* coding region was inserted into the pJIT166-GFP vector by PCR with linker primers (SL-F/R) (Table S1) that contained *SalI* and *BamHI* sites, which generated the C-terminal fusion with the GFP gene under the control of CaMV 35S promoter. All plasmid constructs were confirmed by sequencing. The *35S::GFP* as control and *35S::GbRLK*-*GFP* vectors were transiently expressed in onion epidermal cells using a biolistic particle delivery system (PDS-1000 Bio-Rad, USA). The subcellular localization of the 35S::*GbRLK-GFP* fusion protein was observed with a confocal laser scanning microscope (LSM 510, Zeiss, Germany).

### Vector construction, plant transformation and transgenic plant selection

The *GbRLK* ORF was obtained by PCR with *Sma*I and *Xba*I linker primers (GbRLK4-F/R) (Table S1) and subcloned into the pBI121 plasmid to construct the expression vector *35S::GbRLK* for plant transformation.

Vectors were introduced into *Agrobacterium tumefaciens* (strain GV3101), and transformed *Arabidopsis* was available by the floral dip method^34^. Homozygosity of transgenic plants was determined by the segregation ratio of the *kanamycin* selection marker and PCR analysis of *NPT*II and 35S-*GbRLK* using the primers NPTII-F/R and 35S-GbRLK (Table S1) complementary to the 35S promoter and GbRLK1-R (Table S1) of antisense *GbRLK* were used for confirmation of *35S::GbRLK* transgenic *Arabidopsis*.

Hypocotyls explants from G. *hirsutum* cultivar W0 were transformed using *Agrobacterium fumefecens*-mediated transformation as described previously^35^. The same procedure was followed to determine homozygosity of transgenic cotton.

### *V. dahliae* biomass quantification in plants

Two-week-old *Arabidopsis* plants were inoculated with *V. dahliae* strain VD8 as described above. After visible symptom detection at 14 d post-inoculation, all above-ground tissues of each *Arabidopsis* genotype were harvested and flash-frozen in liquid nitrogen. The samples were ground to powder, approximately 100 mg of which was used for DNA isolation. qRT-PCR was conducted by ABI7500 PCR machine (Applied Biosystems, USA) with the Q-PCR Core kit for SYBR Green I (Eurogentec Nederland BV, NL). For *V. dahliae* biomass measurement, the internal transcribed spacer region of the ribosomal DNA was targeted to generate a 200 bp amplicon using the fungus-specific ITS1-F primer (5-AAAGTTTTAATGGTTCGCTAAGA-3)^44^ in combination with the *V. dahliae*-specific reverse primer ST-VE1-R (5-CTTGGTCATTTAGAGGAAGTAA-3)^45^. The large subunit of the RuBisCo gene from *Arabidopsis* was used sample equilibration. The average fungal biomass was determined using at least four *Verticillium*-inoculated plants for each genotype, and quantificated in plants as described by Ellendorff Ursula^46^.

### Measurement of anthocyanin and chlorophyll content

Anthocyanin was extracted and quantified as described by Gareth *et al.*^47^ from shoot tissues of 10–15 plantlets 14 days after inoculation with VD. Results are expressed as OD at 530 nm per seedling. Chlorophyll was extracted and measured in triplicate as described by Lichtenthaler^48^.

### Field evaluation of transgenic plants

The transgenic lines and non-transgenic W0 were planted in the filed without inoculated VD to conduct their assessment of agronomic performance at Jiangpu Breed Station, Nanjing Agricultural University (JBS/NAU), in Nanjing, China in the 2014 cotton-growing seasons. The transgenic lines, non-transgenic W0, and the resistant cultivar, *G. barbadense* cv. Hai7124, were grown in pathology nurseries in Xiangqiu Breed Station/Nanjing Agicultural University XBS/NAU in Henan and Xinjiang, China in the 2014 cotton-growing seasons. Four transgenic lines and non-transgenic W0 were sowed in rows with a complete block design with three replicates. Seeds were hand planted directly in three replicate plots at a density of 5 seeds m^–1^ in XBS/NAU, Henan, 25 seeds m^–1^ in Xinjiang and 3 seeds m^–1^ in Jiangsu. Fifteen successive plants were chosen and tagged at mature stage in each plot to investigate the height of plants, number of fruit branch and boll number of per plant. Twenty-five bolls were manually harvested in each plot to investigate the weight of boll and lint percentage. Total cotton yield was assessed by hand picking all harvestable bolls. Plants were scored and classified into five grades as described in our previous report^49^. The *Verticillium* wilt disease index was calculated using the following formula: Disease index = [∑ (Ni × i)/(N × 4)] × 100; i = 0 ~ 4, Ni = plant number of reaction I, N is the total number of investigated plants.

### Sample preparation for sequencing and analysis

Two homozygous transgenic lines, K-6 and K-2, which showed differences in resistance to VD and the WT Col-0 were used for deep-sequencing. Inoculation and sampling of the above-ground parts of seedlings were conducted at 0, 48, 96 and 144 h post inoculation. Mock-inoculated plants which were dipped in sterile water were used as the control. All RNA samples were quantified and examined with the Agilent 2100 bioanalyzer (USA). Extracted RNA samples were selected based on 28S/18S rRNA band intensity (2:1) between 1.6 and 2.0. Library construction, sequencing, data processing and digital tag profiling were as described by Wang *et al.*^50^.

The remaining high quality sequences were mapped to *Arabidopsis thaliana* TIGR reference sequences (TIGR, http://www.tigr.org) using SOAP. For monitoring the mapping events on both strands, the sense and antisense sequences were included in the data collection. We mapped all expressed tags onto a preprocessed database of 17 base-long sequences from TIGR located next to the *Nla* III restriction site, and only one mismatch was allowed. Tags mapping to more than one transcript were excluded from our analysis. When multiple types of tags were aligned to different positions of the same gene, the gene expression levels were represented by the sum of all tags.

Statistical analysis was performed to identify differentially expressed genes between the libraries using a rigorous algorithm^51^. Gene expression was normalized to transcripts per million clean tags. For gene expression variance, the statistical t-test was used to identify genes differentially expressed between libraries. *P*-values were adjusted by the multiple testing procedures described by Benjamini and Yekutieli^52^ by controlling the false discovery rate (FDR). We used a stringent value FDR ≤ 0.0001 and the absolute value of |log_2_Ratio| ≤ 1 as the threshold to judge significant differences in gene expression.

## Additional Information

**How to cite this article**: Jun, Z. *et al.* Overexpression of *GbRLK*, a putative receptor-like kinase gene, improved cotton tolerance to *Verticillium* wilt. *Sci. Rep.* **5**, 15048; doi: 10.1038/srep15048 (2015).

## Supplementary Material

Supplementary Tables and Figures

## Figures and Tables

**Table 1:** The disease symptoms of the transgenic *Arabidopsis* and WT induced by *Verticillium dahliae* isolate VD8 inoculation.

Lines	Fresh weight(g)	Diameter of leaf disc (cm)	Content of chlorophyll (nmol/g)	Content of anthocyanin (nmol/g)	Chlorosis	Dead rate (%)	Disease indexes (%)
CK-N1	0.24 ± 0.05	6.42 ± 0.70	0.49 ± 0.09	28.88 ± 1.58	3	31.1 ± 4.6	56.0 ± 1.0
CK-N2	0.22 ± 0.02	6.51 ± 0.62	0.44 ± 0.05	29.36 ± 2.63	3	33.9 ± 4.3	54.8 ± 2.3
K-2	1.02 ± 0.05^**^	10.03 ± 1.13^**^	1.13 ± 0.08^**^	14.46 ± 0.77^**^	1	2.2 ± 1.7^**^	18.7 ± 1.2^**^
K-3	0.48 ± 0.05^**^	9.63 ± 0.85^**^	0.85 ± 0.09^**^	17.21 ± 0.76^**^	2	6.7 ± 3.0^**^	31.0 ± 2.0^**^
K-5	0.65 ± 0.13^**^	10.44 ± 0.97^**^	0.97 ± 0.05^**^	15.20 ± 0.72^**^	1	5.6 ± 1.7^**^	23.3 ± 3.0^**^
K-6	0.44 ± 0.05^**^	9.29 ± 0.74^*^	0.74 ± 0.06^**^	17.34 ± 0.91^**^	2	9.1 ± 3.4^**^	32.7 ± 2.5^**^
CK-P	1.32 ± 0.09	11.65 ± 1.39	1.39 ± 0.07	9.77±1.70	0.00	0.00	0.00

Four-week-old plants were inoculated by root-dip procedure (5 × 10^7^ conidia ml^−1^) and scored for disease symptoms after 4 weeks. Data represent the mean ± SE (n ≥ 15); similar results were obtained from three independent experiments. CK-N1: wild-type Col-0 inoculated with VD8, CK-N2: the control transformed empty vector pBI121 without GbRLK gene inoculated with VD; CK-P: mock-inoculated wild-type Col-0. Extent of leaf chlorosis was performed as described by Veronese *et al.*^25^. Disease index = [∑ (Ni × i)/(N × 4)] × 100; i = 0 ~ 4, Ni = plant number of reaction i. Significance of the phenotypic results was assessed using Student’s t tests (*P < 0.05, **P < 0.01).

**Table 2:** The disease indexes of the transgenic and non-transgenic cotton induced by *Verticillium dahliae* isolate V991 inoculation.

Lines	Disease index (2011)			Disease index (2012)		
	(A)	(B)	(C)	(A)	(B)	(C)
C-19	23.3 ± 3.0	30.0 ± 2.0**	44.7 ± 2.5**	23.3 ± 3.2*	36.7 ± 1.2**	43.3 ± 4.5**
C-26	13.3 ± 3.0**	18.7 ± 1.2**	31.7 ± 2.0**	27.4 ± 1.1	38.6 ± 2.2**	49.2 ± 2.0**
C-27	24.3 ± 2.9	31.0 ± 2.0**	35.7 ± 2.5**	21.3 ± 1.2**	32.5 ± 0.9**	50.0 ± 3.5**
C-29	25.0 ± 2.0	32.7 ± 2.5*	37.7 ± 2.5**	28.9 ± 1.7	41.8 ± 2.7**	55.2 ± 2.6**
CK1	26.3 ± 1.5	39.7 ± 1.5	56.1 ± 1.0	29.2 ± 1.2	58.7 ± 1.7	73.4 ± 2.7
CK2	25.7 ± 1.2	41.3 ± 1.5	58.0 ± 2.0	30.3 ± 2.1	60.5 ± 1.6	72.4 ± 2.9

The transgenic cotton and W0 with two simples and one heart-shaped leaves were inoculated with *VD* spore suspension (5 × 10^7^) and 20 ml for each pot and scored for disease symptoms after 3 weeks. Data represent the mean ± SE (n ≥ 15); similar results were obtained from three independent experiments. CK1: the control transformed empty vector pBI121without *GbRLK* gene inoculated VD8; CK2: non transgenic plant W0 inoculated with VD8. The reaction of the plant towards the inoculated pathogen was rated on a scale from 0 to 4 modified from Ning *et al.*^49^. Disease index = [∑ (Ni × i)/(N × 4)] × 100; i = 0 ~ 4, Ni = plant number of reaction i. (A) (B) and (C) represent the results 3, 4 and 5 weeks after inoculation, respectively. Disease index was calculated comparing data from CK. Significance of the phenotypic results was assessed using Student’s t tests (*P < 0.05, **P < 0.01).

**Table 3:** The agronomic performance, yield and disease indexes of the transgenic and non-transgenic cotton induced by *Verticillium dahliae* in field with a history of a high incidence.

	Lines	Height (cm)	No. fruit branch per plant	Boll number of per plant	Single boll weight (g)	Lint percent (%)	Seed cotton yield(kg)	Disease indexes (%)	Dead rate (%)
Henan	C-19	102.2 ± 10.3**	8.8 ± 0.8**	11.8 ± 1.6**	3.52 ± 0.4*	39.9 ± 1.3*	141.25 ± 11.6**	40.9 ± 5.6**	29.1 ± 4.7**
	C-26	105.6 ± 9.1**	9.4 ± 1.9**	12.4 ± 3.6**	3.32 ± 0.3*	38.4 ± 1.6	143.15 ± 12.9**	20.6 ± 3.3**	31.7 ± 1.9**
	C-27	105.6 ± 7.6**	9.2 ± 1.2**	11.4 ± 2.2**	3.43 ± 0.3*	38.8 ± 2.5	141.05 ± 16.9**	33.0 ± 4.7**	18.2 ± 2.5**
	C-29	104.7 ± 6.6**	8.6 ± 1.1**	11.6 ± 1.5**	3.34 ± 0.2*	36.5 ± 2.2	139.36 ± 11.6**	32.8 ± 2.9**	21.4 ± 3.1**
	W0	86.3 ± 7.5	6.5 ± 0.8	7.6 ± 1.1	2.97 ± 0.4	37.5 ± 2.4	83.58 ± 9.3	65.6 ± 6.3	54.8 ± 6.3
	H7124							10.7 ± 2.8	0.00
Xinjiang	C-19	66.2 ± 5.9**	9.4 ± 0.58**	6.4 ± 1.3**	3.86 ± 0.2*	43.0 ± 2.0	437.5 ± 63.4**	50.1 ± 8.2**	31.2 ± 2.9**
	C-26	67.8 ± 3.7**	9.6 ± 1.0**	6.0 ± 0.9**	3.95 ± 0.6*	44.2 ± 1.9	421.9 ± 40.1**	60.8 ± 6.7**	35.0 ± 6.1**
	C-27	65.4 ± 4.2**	9.2 ± 0.8**	6.8 ± 0.8**	4.00 ± 0.4*	43.2 ± 1.1	484.2 ± 41.9**	36.4 ± 3.4**	33.6 ± 4.5**
	C-29	67.4 ± 6.1**	8.9 ± 1.1**	6.2 ± 1.1**	3.78 ± 0.7*	42.5 ± 2.3	417.2 ± 21.9**	43.8 ± 3.9**	36.3 ± 2.8**
	W0	48.5 ± 2.7	7.6 ± 0.32	4.3 ± 0.8	3.26 ± 0.3	42.6 ± 2.1	249.5 ± 24.9	94.6 ± 3.9	66.5 ± 7.5
	H7124							29.9 ± 2.1	0.00

The transgenic lines, non-transgenic W0, and the resistance variety *G. barbadense* cv. Hai7124 were grown at a farm in Henan and Xjinjiang province, China in the 2014 cotton-growing seasons. Seed was planted in a field with a history of a high incidence of *Verticillium* wilt caused by *Verticillium dahliae*. Data represent the mean ± SE (n ≥ 15); similar results were obtained from three independent experiments. Plants were scored and classified into five grades as described in our previous report^49^. The *Verticillium* wilt disease index was calculated using the following formula: Disease index = [∑ (Ni × i)/(N × 4)] × 100; i = 0 ~ 4, Ni = plant number of reaction I, N is the total number of investigated plants. The analysis of significance was calculated comparing data from W0. Significance of the phenotypic results was assessed using Student’s t tests (*P < 0.05, **P < 0.01).
