# Supplementary material for: Overexpression of GbRLK, a putative receptor-like kinase gene, improved cotton tolerance to Verticillium wilt
Source: Sci Rep. 2015 Oct 8;5:15048. doi: 10.1038/srep15048 (PMC4597213; doi:10.1038/srep15048)
Supplement: Supplementary Tables and Figures [file srep15048-s1.pdf]

Title:

Overexpression of *GbRLK*, a putative receptor-like kinase gene, improved cotton tolerance to *Verticillium* wilt

Journal name: ***Scientific Reports***

Jun Zhao, Zhiyuan Zhang, Yulong Gao, Lei Zhou, Lei Fang, Xiangdong Chen, Zhiyuan Ning, Tianzi Chen, Wangzhen Guo, Tianzhen Zhang*

National Key Laboratory of Crop Genetics & Germplasm Enhancement, MOE Hybrid Cotton R&D Engineering Research Center, Nanjing Agricultural University, Nanjing 210095, Jiangsu province, China.

***** Correspondence and requests for materials should be addressed to Tianzhen Zhang (cotton@njau.edu.cn).

Supporting Information

Table S1. The primers used in the research.

Table S2. The agronomic performance and yield of the transgenic and non-transgenic.

Table S3. The primers for validation the results of profile and the efficiency of amplification.

Table S4. Expression value (TPM) of specially genes in different samples.

Table S5. Biological process analysis of differential up-regulated transcription factors in transgenic and non-transgenic *Arabidopsis.*

Table S6. Gene ontology analyses of up-regulated genes at different time.

Table S7. Biological process analysis of differential up-regulated genes in transgenic and non-transgenic *Arabidopsis* after inoculation.

Figure S1. Gene expression and Southern blotting analysis of *GbRLK*.

Figure S2. The prediction result of conserved domain of GbRLK protein from At and Dt sub-genomes.

Figure S3. Molecular analysis of the independent transgenic *Arabidopsis* lines.

Figure S4. PCR tests of transgenic plants of the independent cotton transgenic lines.

Supporting Tables

Table S1 The primers used in the research

GbRLK1-F	ATGGATAAATTCCTGAACGAA
GbRLK1-R	ATAAATTGTG- ACCATTTCAATT
SL-F	GTCGACATGGATA-AATTCCTGAACGAA
SL-R	GGATCCATAAATTGTGACCATTTCAATT
GbRLK2-F	CAATAGGCACTGCTAAAGGG
GbRLK2-R	ACCACGATGCTCATCAAAGG
EF-F	AGACCACCAAGTACTACTGCAC
EF-R	CCACCAATCTTGTACACATCC
AtRuBisCo-F3	GCAAGTGTTGGGTTCAAAGCTGGTG
AtRuBisCo-R3	CCAGGTTGAGGAGTTACTCGGAATGCTG
GbRLK3-F	GCCAAGAATGGTTTCCAAGAT
GbRLK3-R	ACATTTTGTATTCTGGAAG CACA
NPTII-F	CACCCTATTCCC CTATCACTC
NPTII-R	TACAACCCCATCCCCTCCCA
GbRLK4-F	CGTCTAGATAGTGTTTGCCCAGAAACTC
GbRLK4-R	ATCCCGGGTATTCTGGAAGCACATTA
35S-GbRLK	CACAATCCCACTATCCTTCG
ITS1-F	AAAGTTTTAATGGTTCGCTAAGA
ST-VE1-R	CTTGGTCATTTAGAGGAAGTAA
**Special primers for amplification in sub-genome A and D**	
SP-A1-F	AGAGCTCTTGTATACGAGTACATGGTAAATGGTTTA
SP-A1-R	CATTTATGTGTAATAGGATATGGCATCCAAAGCTC
SP-A2-F	ACTAGGCACTGCTAAAGGGATTGCTTACTTGTAT
SP-A2-R	ATCAAGGTTCTTCTCTTACCAATGATCTCAAACAA
SP-D1-F	TACCTGAAAGCCAAGAATGGTTTCCAAGAT
SP-D1-R	GACAGGAGTTGCATGTATTGAAGCGGAG
SP-D2-F	AATCATGTGGAGTTAATGATGGTCTGCGTAA
SP-D2-R	TTTCATGACAGGAGTTGCATGTATTGAAGC

AP: Adapter Primer; AUAP: Abridged Universal Amplification Primer. The underlined sequence represent the restriction enzyme sites.

Table S2 The agronomic performance and yield of the transgenic and non-transgenic

Lines	Height(cm)	No. fruit branch per plant	Boll number of per plant	Single boll weight (g)	Lint percent (%)	Seed cotton yield(kg)
C-19	117.2±7.1	14.2±1.8	17.3±1.9	3.81±0.4	38.2±0.9	197.54±8.9
C-26	115.9±5.1	14.0±2.1	16.9±1.6	3.89±0.5	37.9±1.8	203.24±7.6
C-27	117.3±4.6	13.9±0.9	17.4±2.0	3.91±0.5	39.1±1.7	201.97±10.8
C-29	115.8±6.8	14.4±1.1	16.8±1.4	3.84±0.3	38.1±2.0	199.67±6.6
W0	116.9±8.5	14.2±1.0	17.6±1.2	3.87±0.6	38.6±1.9	200.58±11.5

The transgenic lines, non-transgenic W0 were grown at a farm in Jiangsu province, China in the 2014 cotton-growing seasons. Seed was planted in a field without diseased soil. Data represent the mean ± SE (n≥15); similar results were obtained from three independent experiments.

Table S3 The primers for validation the results of profile and the efficiency of amplification

Gene Identifier	Sequence of primer	Efficiency of amplification	Correlation coefficient
AT2G41240.1	F:GACCAAAACAGTAAGTCAGAGG<br>R:GAAGATGAAGCACCCACAA	96.60%	0.993
AT3G56970.1	F:TTGGGAGTATCTGTTTCGTCA<br>R:TAGCCTCTTCACTTGCTGTTG	95.10%	0.992
AT5G04150.1	F:GAACGAGACCGCCGTAGAAA<br>R:ACCATTGATGAGTTCCTCCAGTC	94.60%	0.991
AT3G56980.1	F:TGTTTCTGTTTCGTCGGAGGG<br>R:TGAGATTTGGACCATCACTTCG	92.90%	0.997
AT5G45820.1	F:CAGTGATGCGTCTCGTTCGT<br>R:CTCCATCATAACCTTTCTTGCCTA	95.90%	0.993
AT1G13609.1	F:GAATGCTTAAAGAACATTGCCTGTG<br>R:AACGGTTGGATTGTTGGTTTCTG	91.70%	0.997
AT4G25470.1	F:CAGGCGGTGATTACAGTCC<br>R:ATTTCCTTGGCACAGGTTGATT	98.60%	0.997
AT1G74930.1	F:TGGTGAAGCAAGCGATGAAG<br>R:TCGGGAGTGTCGTAAGAGCC	97.50%	0.998
AT5G57560.1	F:CACAAGGCAAAGGAGACAAA<br>R:AAGCCACGGTAAGAAGCAGT	93.20%	0.999
AT1G35140.1	F:GATTCGCTTACATCTGGGTTGG<br>R:TCACCATCCCGTCTAAACCAA	97.50%	0.998
AT5G59310.1	F:CCATCTCCACGAGCACCAAC<br>R:CGAAACCATACTCTTCAGGCAAA	88.80%	0.997
AT1G21910.1	F:ACTTCATCAGCCGTCTCGT<br>R:GTATTATGGACCGTCGGATTA	91.10%	0.997
AT4G22120.1	F:AACCCAACCCAATGGCTGAC<br>R:TGCGGATTGATTGAGGAAAG	95.00%	0.993
AT5G59320.1	F:GGCTTTCGCTTTGAGGTTCTT<br>R:GTTGGCGGTCTGGTGTGGTT	101.40%	0.993
AT1G65590.1	F:ATCCCGACATTATTTGCCAC<br>R:TGTATCGCTGAGAGGAAGAGTA	95.50%	0.998

Table S4 Expression value (TPM) of specially genes in different samples

Gene identifier	1	2	3	4	5	6	7	8	9	10	11	12	13	14	15	16	17	18	19	20	21	Description
AT3G49620	0	12.6	1.37	19.6	9.15	3.11	1.64	18.5	6.78	15.7	13.5	8.19	9.98	16.7	21.9	5.01	66.4	28.6	3.83	17	11.1	DIN11
AT2G41240	0	103	136	58.1	9.34	7.89	13.5	10.1	73.2	41.6	23.1	30	17.9	0	18.9	97.8	17.1	29.2	3.43	7.39	11.9	BHLH100 transcription factor
AT3G56970	0	102	119	128	1.45	1.23	5.75	7.57	58.2	52.6	18.9	6.31	8.67	1.01	15.3	103	46.2	68.5	15.7	22.4	12.4	BHLH038
AT5G04150	0	22.4	21.8	13.1	1.04	1.59	8.2	12.4	13	29.9	1.72	16.8	12.2	0.6	11.8	17.6	38	14.3	7.06	10.6	11.3	BHLH101
AT3G56980	0	84.8	133	107	1.63	1.42	6.2	4.21	52.2	32.6	14.7	3.72	8.29	0	11.6	78.8	33.7	36.9	5.65	7.79	21.7	BHLH039
AT5G45820	0	9.55	11	5.85	8.76	11.2	6.15	11.4	3.91	10.5	1.33	7.98	7.94	1.21	9.92	9.82	8.01	7.65	25.6	7.99	8.14	CIPK20
AT3G13404	0	2.03	0.39	0.61	0.97	2.91	0	10.9	1.03	1.24	0.38	2.52	1.02	1.41	7.82	1.88	0	1.21	1.01	0.6	0	unknown protein
AT1G13609	0	25.6	60	46.4	3.11	3.53	5.79	3.79	13.2	21.7	5.53	2.33	1.75	0	7.06	21.7	32.7	13.5	2.62	5.59	10.9	Encodes a defensin-like family protein
AT4G34410	0	5.29	9.05	5.04	3.7	2.66	2.67	4.21	24.3	12.1	11.8	8.4	4.68	0.4	5.34	12.5	13.5	1.81	3.43	1.4	1.04	RRTF1
AT4G25470	0	7.12	21.2	3.43	5.84	4.78	1.85	5.47	10.7	16.8	4.38	7.56	10.2	1.81	4.77	9.4	28.7	3.63	8.9	3	1.46	CBF2
AT2G15780	0	3.05	18.1	6.25	4.67	0.78	4.1	5.68	2.26	14.2	4	0	0	0.6	4.58	0.63	17.7	9.47	0	0.59	0.43	Gycine-rich protein
AT4G16740	0	2.24	0.98	0.61	0.39	1.04	0.41	2.94	0	1.24	0	0.42	1.43	0.4	4.2	0.42	0	0.6	0	0.4	1.46	ATTPS03
AT5G18420	0	7.52	22.5	3.63	1.95	2.49	28.7	22.9	7.81	2.06	4.57	5.53	6.97	8.25	19.1	4.6	7.38	3.63	3.43	1.6	2.92	Unknown protein
AT1G74890	0	1.42	1.18	1.21	1.56	3.53	1.23	3.16	1.85	3.3	2.29	1.47	0.41	0.81	3.24	2.3	0	1.21	2.22	1.6	2.51	ARR15
AT4G27654	0	3.46	0.39	4.64	2.4	0.11	0.62	7.36	14.6	22.3	5.34	2.73	1.83	0.4	3.05	6.89	17.5	2.22	1.82	1.6	3.76	Unknown protein
AT1G59930	0	2.24	0.98	1.82	0.78	0.83	0	3.37	1.23	1.03	0	1.26	1.63	0.6	3.05	2.09	0.42	1.01	1.21	0.8	0	Unknown protein
AT1G02820	0	2.24	1.18	1.21	0.97	0	3.69	2.94	2.88	2.27	0	2.1	2.24	0.4	2.86	1.25	2.95	1.41	3.23	1.2	1.46	LEA3 family protein
AT4G29610	0	2.24	1.18	5.04	0.11	0.49	0.15	6.94	14.1	13.9	4.19	1.89	2.24	2.21	2.48	7.94	5.27	1.81	3.43	3.39	3.97	Cytidine deaminase

Note: The number 1 - 4, 8 -11 and 15 -18 represent the different period samples (0, 48, 96 and 144 h) after inoculation *Verticillium* of Col-0, K-2 and K-6, respectively.

The number 5-7, 12-14 and 19-21 represent the different period samples (48, 96, and 144h) after inoculation water as control of Col-0, K-2 and K-6, respectively.

Table S5 Biological process analysis of differential up-regulated transcription factors in transgenic and non-transgenic *Arabidopsis.*

Gene ID	Description	Biological process
	**Disease**	
AT2G17040	NAC domain containing protein 36	Leaf morphogenesis, negative regulation of cell size, response to chitin
AT4G34410	RRTF1, ethylene-responsive transcription factor ERF109	Defense response to fungus, response to chitin
AT1G74930	Ethylene-responsive transcription factor ERF018	Response to biotic stimulus, wounding
AT4G17490	Ethylene-responsive transcription factor	Ethylene mediated signaling pathway, response to chitin
AT1G51700	Dof zinc finger protein DOF1.7	Response to chitin
AT3G50060	MYB77	Lateral root development, response to chitin, ethylene stimulus and salicylic acid stimulus
	**Water deprivation**	
AT4G25470	Dehydration-responsive element-binding protein	Response to cold and dehydration
AT1G09200	Histone H3	Methylation, dehydration stress
AT2G41240	Transcription factor bHLH100	Drought, Iron deficiency stress
AT1G22190	Ethylene-responsive transcription factor ERF058	Response to osmotic, cold, drought stress
AT4G27410	RD26, NAC domain-containing protein 72	Response to abscisic acid stimulus and water deprivation, multicellular organismal development
	**Water deprivation and disease**	
AT1G21910	Ethylene-responsive transcription factor ERF012	Cellular response to freezing, heat, drought, jasmonic acid and salicylic acid stimulus
AT1G71030	MYBL2	Anthocyanin biosynthetic process, response to abscisic acid, salt stress, jasmonic acid, salicylic acid and ethylene stimulus
AT5G67300	MYBR1, transcription factor MYB44	Response to chitin, abscisic acid, dehydration, salt stress, jasmonic acid, salicylic acid and ethylene stimulus
AT4G37260	MYB73 myb domain protein 73	Response to chitin, abscisic acid, jasmonic acid, salicylic acid and ethylene stimulus.
	**Other**	
AT3G56970	BHLH038/ORG2	Iron deficiency-mediated stress
AT5G04150	Transcription factor bHLH101	Iron deficiency-mediated stress
AT3G56980	BHLH039/ORG3	Iron deficiency-mediated stress
AT5G25190	Ethylene-responsive transcription factor ERF003	Ethylene mediated signaling pathway

AT3G59060	Transcription factor PIF5	Ethylene biosynthetic process
AT3G56940	CRD1	Oxidation-reduction process, chlorophyll biosynthetic process, photosynthesis
AT3G59080	Aspartyl protease family protein	Peptidolysis
AT5G50915	Transcription factor bHLH137	Response to gibberellin stimulus
AT2G37025	TRFL8 protein TRF-like 8	
AT1G14440	Homebox protein 21	
AT2G42380	BZIP34	
AT2G23290	MYB70	
AT5G08330	Transcription factor TCP21	
AT4G36570	Protein RAD-like 3	
AT3G58120	BZIP61	
AT1G25550	Myb-like transcription factor-like protein	
AT5G35970	Putative DNA-binding protein	
AT5G04760	Duplicated SANT DNA-binding domain-containing protein	

Table S6 Gene ontology analyses of up-regulated genes at different times

GO categories	Count	*p*-Value
**48h up-regulated genes (198)**		
Regulation of transcription, DNA-dependent	41	1.13×10^{-8}
Regulation of transcription	37	5.91×10^{-4}
Oxidation reduction	22	4.24×10^{-3}
Protein folding	13	2.27×10^{-6}
Response to abscisic acid stimulus	13	4.30×10^{-6}
Response to cadmium ion	13	1.07×10^{-5}
Response to salt stress	13	3.56×10^{-5}
Response to gibberellic acid stimulus	12	3.55×10^{-9}
Response to heat	11	4.25×10^{-8}
Response to jasmonic acid stimulus	11	3.88×10^{-7}
Response to auxin stimulus	11	3.53×10^{-4}
Response to salicylic acid stimulus	9	9.82×10^{-6}
Response to ethylene stimulus	7	1.08×10^{-3}
Response to cold	6	2.07×10^{-2}
Oligopeptide transport	5	1.11×10^{-3}
Response to hydrogen peroxide	5	4.49×10^{-3}
Response to chitin	5	7.66×10^{-3}
Ethylene mediated signaling pathway	5	1.94×10^{-2}
Leaf development	5	2.19×10^{-2}
Lipid catabolism	5	4.51×10^{-2}
Response to high light intensity	4	9.10×10^{-4}
Abscisic acid mediated signaling	4	8.64×10^{-3}
Transmembrane receptor protein tyrosine kinase signaling pathway	4	2.63×10^{-2}
Lipid transport	4	4.72×10^{-2}
**96h up-regulated genes (26)**		
dTDP biosynthesis	2	3.39×10^{-6}
Translational elongation	2	5.01×10^{-4}
'de novo' IMP biosynthesis	1	6.07×10^{-3}
Protein folding	2	2.16×10^{-2}
Folic acid and derivative biosynthesis	1	2.41×10^{-2}
Response to cadmium ion	2	2.82×10^{-2}
RNA modification	1	4.97×10^{-2}
**1446h up-regulated genes (3)**		

Respiratory burst	1	3.18×10^{-4}
Rhythmic process	1	3.45×10^{-3}
Mitochondrial transport	1	3.45×10^{-3}
Hydrogen peroxide catabolism	1	4.51×10^{-3}
Defense response to fungus	1	5.51×10^{-3}
Response to oxidative stress	1	1.65×10^{-2}

Table S7 Biological process analysis of differential up-regulated genes in transgenic and non-transgenic Arabidopsis after inoculation

Biological Process	Gene ID
Related to jasmonic acid	AT5G37260; AT3G09600; AT5G60890; AT4G38620; AT5G13930; AT5G59780; AT5G61420; AT3G55730; AT4G26850; AT4G01060
Related to abscisic acid	AT1G75750; AT5G37260; AT3G09600; AT5G60890; AT4G38620; AT4G22200; AT5G61420; AT3G55730; AT1G63840; AT1G32640; AT4G01060; AT2G36530; AT1G72770; AT4G21670; AT2G28350; AT2G04240; AT2G18790
Related to salicylic acid	AT5G37260; AT5G67160; AT3G09600; AT5G60890; AT4G38620; AT5G59780; AT5G61420; AT3G55730; AT4G01060
Response to salt stress	AT5G37260; AT3G09600; AT5G60890; AT4G38620; AT2G04240; AT5G59780; AT3G55730; AT3G15950; AT4G01060; AT5G14800; AT2G36530
Related to anthocyanin biosynthesis	AT5G13930;AT2G28350
Oxidation reduction	AT1G66540; AT5G21100; AT2G34490; AT4G37320; AT3G30180; AT5G53090; AT3G27060; AT3G59890; AT4G25700; AT4G39510; AT3G28740; AT2G37760; AT5G63910; AT4G29720; AT3G20100; AT5G14800; AT1G23020
Response to hydrogen peroxide	AT2G19310; AT3G17790; AT4G12400; AT3G12580
Response to chitin	AT4G34410; AT2G04240; AT5G59780; AT1G32640; AT3G46620
Callose deposition during defense response	AT1G24100; AT4G26850
Related to ethylene	AT5G37260; AT3G09600; AT4G38620; AT5G59780; AT1G70560; AT3G55730; AT1G19210; AT4G34410; AT5G21960; AT1G74930; AT2G44940
Tyrosine kinase signaling pathway	AT5G14210; AT5G67280; AT5G56040; AT1G63430
Sodium ion transport	AT3G51860; AT5G64280
Response to drought	AT2G41240; AT4G25480; AT4G25490; AT1G12110
Response to biotic	AT4G12470; AT4G34410; AT5G02490; AT5G61420; AT5G02500
Response to heat	AT2G19310; AT5G02490; AT4G26850; AT4G12400; AT5G56010; AT3G12580; AT5G14800; AT5G02500; AT3G09440

Supplementary Figures

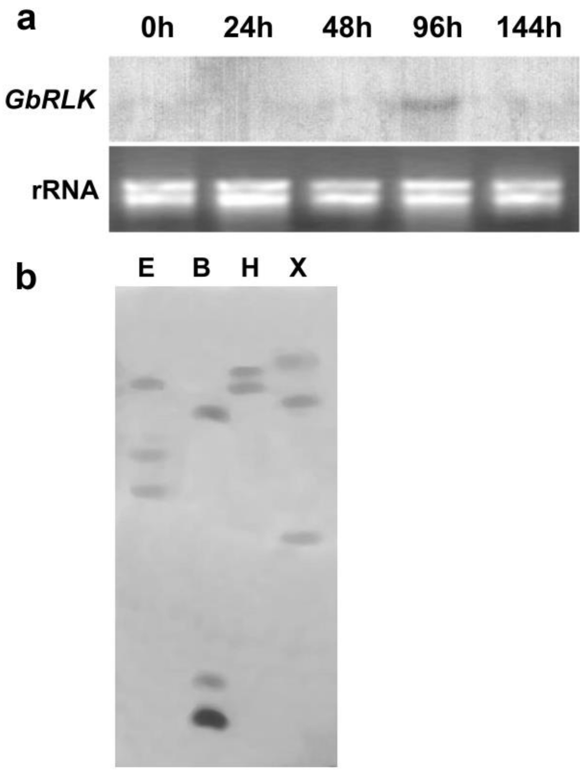

Figure S1. Gene expression and Southern blotting analysis of *GbRLK*.

(a) Northern blotting of *GbRLK* expression following inoculation with VD at different time intervals.

(b) Southern blotting analysis of *GbRLK*. E: *Eco*RI; B: *Bam*HI; H: *Hin*dIII; X: *Xba*I.

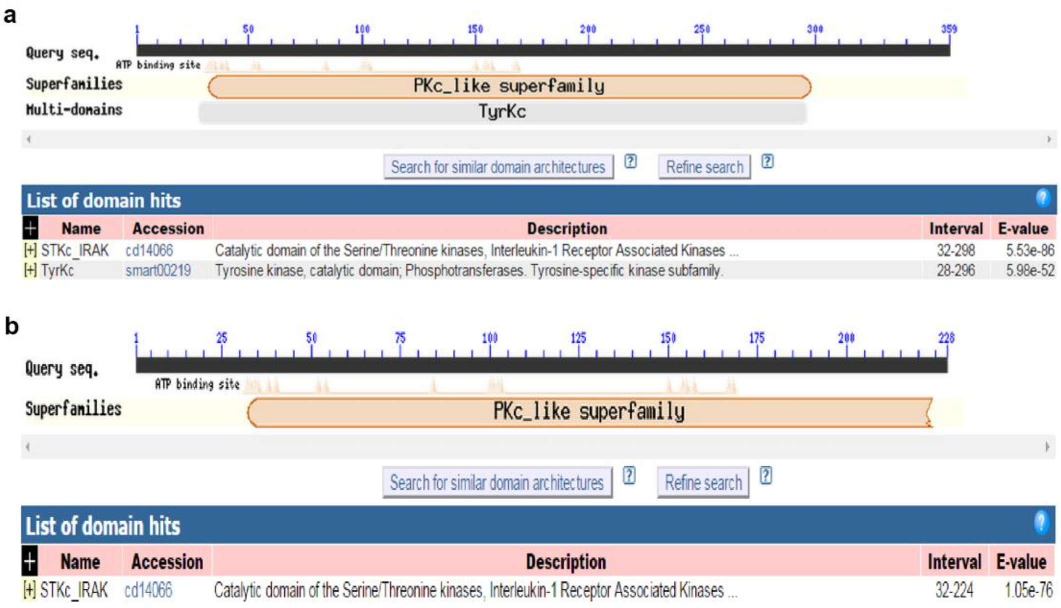

Figure S2. The prediction result of conserved domain of GbRLK protein from At and Dt sub-genomes. (**a**) The prediction result of conserved domain of GbRLK protein from Dt subgenome. (**b**) The prediction result of conserved domain of GbRLK protein from At sub-genomes.

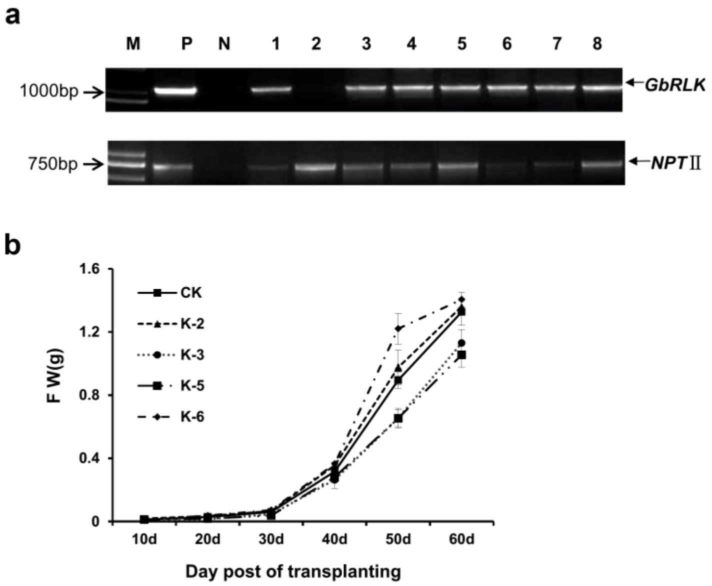

Figure S3. Molecular analysis of the independent transgenic *Arabidopsis* lines.

(a) PCR tests of transgenic plants

(b) Fresh weight of transgenic and non-transgenic *Arabidopsis* at different growth times.

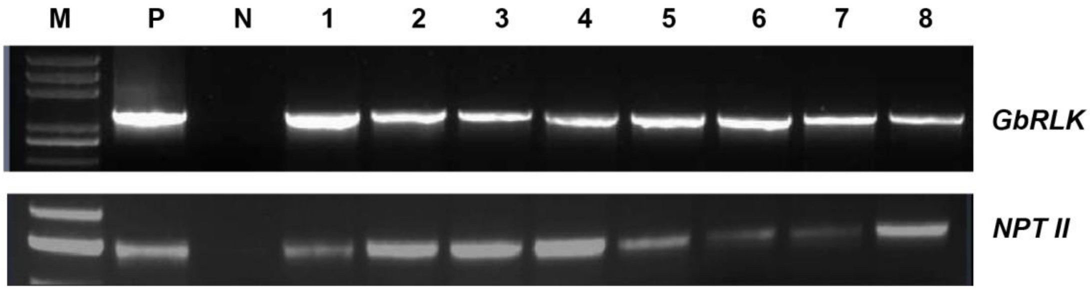

Figure S4. PCR tests of transgenic plants of the independent cotton transgenic lines.
